# Significant differences in femoral torsion values depending on the CT measurement technique

**DOI:** 10.1007/s00402-016-2536-3

**Published:** 2016-08-08

**Authors:** Peter Kaiser, René Attal, Mark Kammerer, Michael Thauerer, Lea Hamberger, Raul Mayr, Werner Schmoelz

**Affiliations:** 1 Department of Trauma Surgery, Medical University of Innsbruck, Anichstrasse 35, 6020 Innsbruck, Austria; 2 Department of Radiology, Medical University of Innsbruck, Innsbruck, Austria

**Keywords:** Femoral, Torsion, Anteversion, Patella dislocation, Derotational osteotomy

## Abstract

**Introduction:**

This study compared the feasibility of six different CT-based measurement techniques for establishing an indication for derotational osteotomy in the cases of patellar instability or femoral fracture.

**Materials and methods:**

CT scans of 52 single human cadaver femora were measured using six different torsion measurement techniques (described by Waidelich, Murphy, and Yoshioka on transverse images and Hernandez, Jarrett, and Yoshioka on oblique images). All measurements were performed by four observers twice to assess intraobserver and interobserver agreement. The intraclass correlation coefficient (ICC), ANOVA, and Bonferroni post hoc test were used for the statistical analysis.

**Results:**

Significant differences (*P* < 0.001) between the values for femoral torsion were observed with all techniques except Yoshioka’s techniques on transverse and oblique slices (*P* = 1.000) (transverse images: Waidelich 22.4° ± 6.8°, Murphy 17.5° ± 7.0°, Yoshioka 13.4° ± 6.9°; oblique images: Hernandez 11.4° ± 7.4°, Jarrett 14.9° ± 7.5°, Yoshioka oblique 13.4° ± 7.1°). Intraobserver and interobserver agreement showed a high level of reproducibility (ICC 0.877–0.986; mean 0.8°–2.9°) for all techniques, with the greatest difference being observed with Hernandez’s technique (11.4°/10°).

**Conclusions:**

Femoral torsion values depend on the measurement technique. When derotational osteotomy is being considered, it is essential to use different threshold values depending on the measurement technique.

## Introduction

Femoral torsion, also known as femoral rotation or femoral version, refers to the twist between the proximal and distal parts of the femur on the transverse plane. Various imaging techniques, including radiography [9], ultrasound [4], low-dose biplanar radiography [17], computed tomography (CT) [8, 11–13, 18, 24, 25], and magnetic resonance imaging (MRI) [3, 20, 23] have been used to assess femoral torsion. With their speed, precision, and ease of use, cross-sectional imaging modalities, such as CT or MRI, are regarded as the gold standard for measuring torsion in the femur.

Descriptions of various measurement techniques have been published, using transverse or oblique and single or superimposed image slices. The techniques also use different anatomical landmarks for measurement. As a result, a wide range of the standard values for femoral torsion (7°–24.1° internal torsion) has been reported in the literature [3, 5, 14, 15, 20, 21, 23–25].

The assessment of femoral torsion is important in the cases of maltorsion after a femur fracture or in the cases of lateral patellar instability, as an excessive femoral internal torsion has been described as a risk factor [2, 6, 7, 12, 19, 22]. In the cases of recurrent patellar instability, femoral internal torsion of more than 15°–25° is considered to represent an indication for derotational femoral osteotomy [1, 2, 12].

The threshold range for conducting a derotational osteotomy overlaps with the range of the standard values for femoral torsion. It is, therefore, possible that patients with recurrent patellar instability in whom femoral torsion lies within the standard range might also be regarded as candidates for derotational osteotomy. As there are multiple measurement techniques, the influence of the technique on the value measured and thus on the threshold value remains unclear. Influencing factors include the use of different anatomical landmarks for measurement and high levels of intraobserver and interobserver agreement.

The purpose of the present study was, therefore, to evaluate the differences in femoral torsion values that arise due to different CT measurement techniques and the associated intraobserver and interobserver agreement. The findings may be helpful for surgeons who use CT values to establish the indication for femoral derotational osteotomy.

## Materials and methods

CT scans of 26 pairs of human cadaver femora (11 female, 15 male) were used for CT measurement of femoral torsion. All femora were dissected leaving just the femoral bone itself. The donors’ mean age was 73.7 years (range 51–90 years). The donors gave informed consent for medical studies to the anatomical institute during lifetime.

A LightSpeed VCT (GE Healthcare, Little Chalfont, UK) was used for scanning, with scan properties of 100 kV and 9 mAs. The specimens were positioned with their longitudinal axis along the CT bench for scanning. The scans were all reformatted to first transverse slices with a slice distance of 2.5 mm and a slice thickness of 2.5 mm; and second, oblique slices parallel to the femoral neck with a slice distance of 5 mm and a slice thickness of 5 mm. Measurements were performed with the Impax EE R20 viewer (Agfa Healthcare, Mortsel, Belgium).

Femoral torsion was independently measured using six different measurement techniques by four observers (two trauma surgeons and two radiologists) for the assessment of the interobserver agreement. All the measurements were repeated after a period of 8–12 weeks for the calculation of the intraobserver agreement. All observers were taught all measurement techniques in advance with different torsion CT images. The applied measurement techniques of femoral torsion are described in the literature and commonly used in various clinics assessed through conversation with colleagues. No anatomical analysis of the femoral torsion with a reference measurement technique, such as a goniometer or 3D surface digitizing with volume rendering, was used for validation, because the measured femoral torsion will always depend on the definition of landmarks and reference points independent of the measurement technique (goniometer, 3D surface digitizing, CT scan, etc.).

Femoral torsion was assessed by the angle between axes in the proximal and distal parts of the femur. For all the techniques, the axis in the distal part of the femur was a tangent to the posterior condyles on a single slice of a transverse image in which the condyles had their maximum expansion from anterior to posterior (Fig. 2h) [18].

**Fig. 1:**
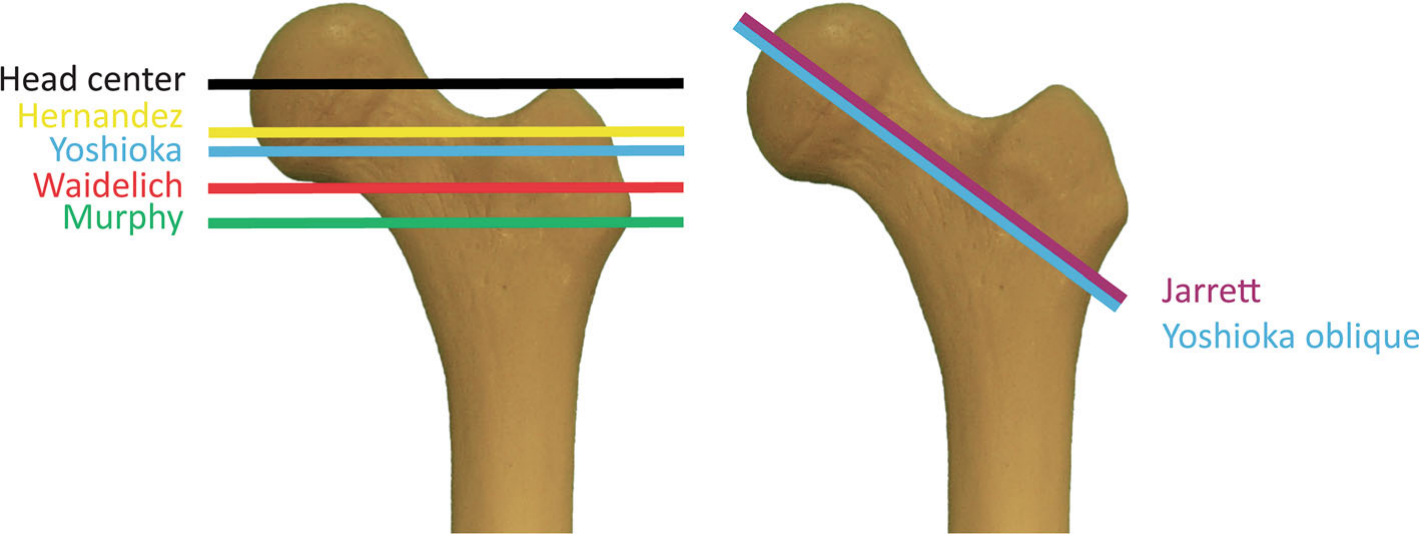
Level and orientation of computed tomography slice selection (*left,* transverse slices; *right,* oblique slices)

**Fig. 2:**
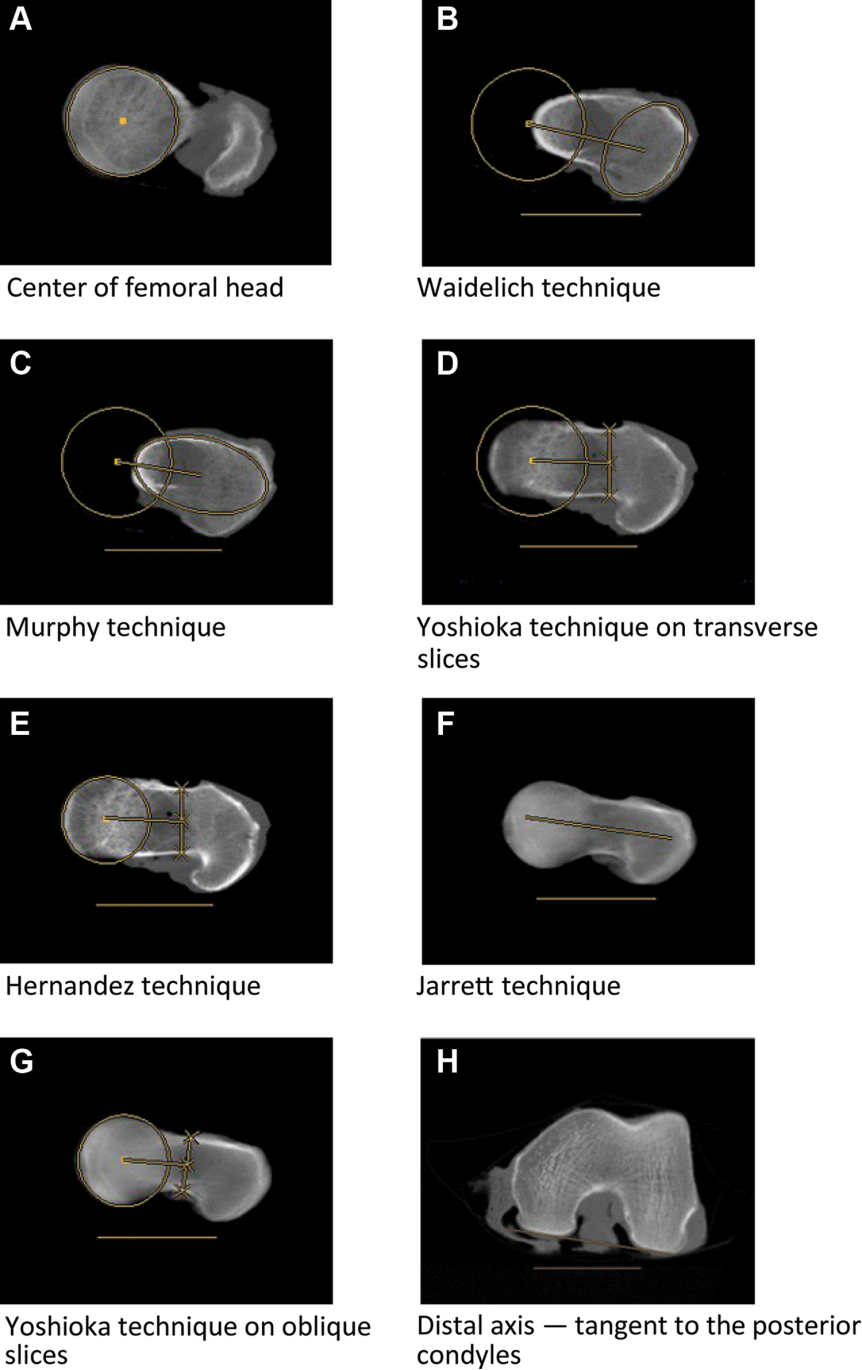
Measurement techniques (transverse slices: **a**–**e**, **h**; oblique slices: **f**, **g**)

Six different techniques for measuring the axis in the proximal part of the femur were used, in accordance with the following descriptions (Figs. 1, 2):

1. The technique described by Waidelich et al. on superimposed transverse slices [24]: the center of the femoral head on one transverse slice was connected to the center of an ellipse around the greater trochanter on another transverse slice that was located between the tip of the major trochanter and the minor trochanter.
2. The technique described by Murphy et al. on superimposed transverse slices [12, 18]: the center of the femoral head on one transverse slice was connected to the center of an ellipse around the base of the femoral neck on another transverse slice.
3. The technique described by Yoshioka et al. on superimposed transverse slices [25]: the center of the femoral head on one transverse slice was connected to the center of the femoral neck at its narrowest width on another transverse slice.
4. The technique described by Hernandez et al. on a single transverse slice [11]: the center of the femoral head was connected to the center of the femoral neck on a single transverse slice. A slice was chosen in a location in which the femoral head, femoral neck, and major trochanter were visible.
5. The technique described by Jarrett et al. on a single oblique slice [13]: a line parallel to the femoral neck represented the proximal axis on a single oblique slice.
6. The technique described by Yoshioka et al. on superimposed oblique slices [25]: the center of the femoral head on one oblique slice was connected to the center of the femoral neck at its narrowest width on another oblique slice.

All the observers were initially instructed in these CT measurement techniques on different single-femur CT scans in advance, before the data were recorded.

The data were analyzed using IBM SPSS Statistics for Windows, Version 21.0 (Armonk, New York, USA: IBM Corporation). Descriptive values, analysis of variance (ANOVA) for repeated measurements, and the Bonferroni post hoc test were used to analyze differences between the six measurement techniques. All the measurements were included in the calculation in the comparison of the six techniques to eliminate intraobserver and interobserver agreement. Intraobserver and interobserver agreement was analyzed using the intraclass correlation coefficient (ICC) and descriptive data. The scoring system presented by Fleiss et al. [10] was used to analyze the results (ICC > 0.75 good, 0.4–0.75 fair, <0.4 poor). The significance level was set at *P* < 0.05.

## Results

### Comparison of the measurement techniques

**Fig. 3:**
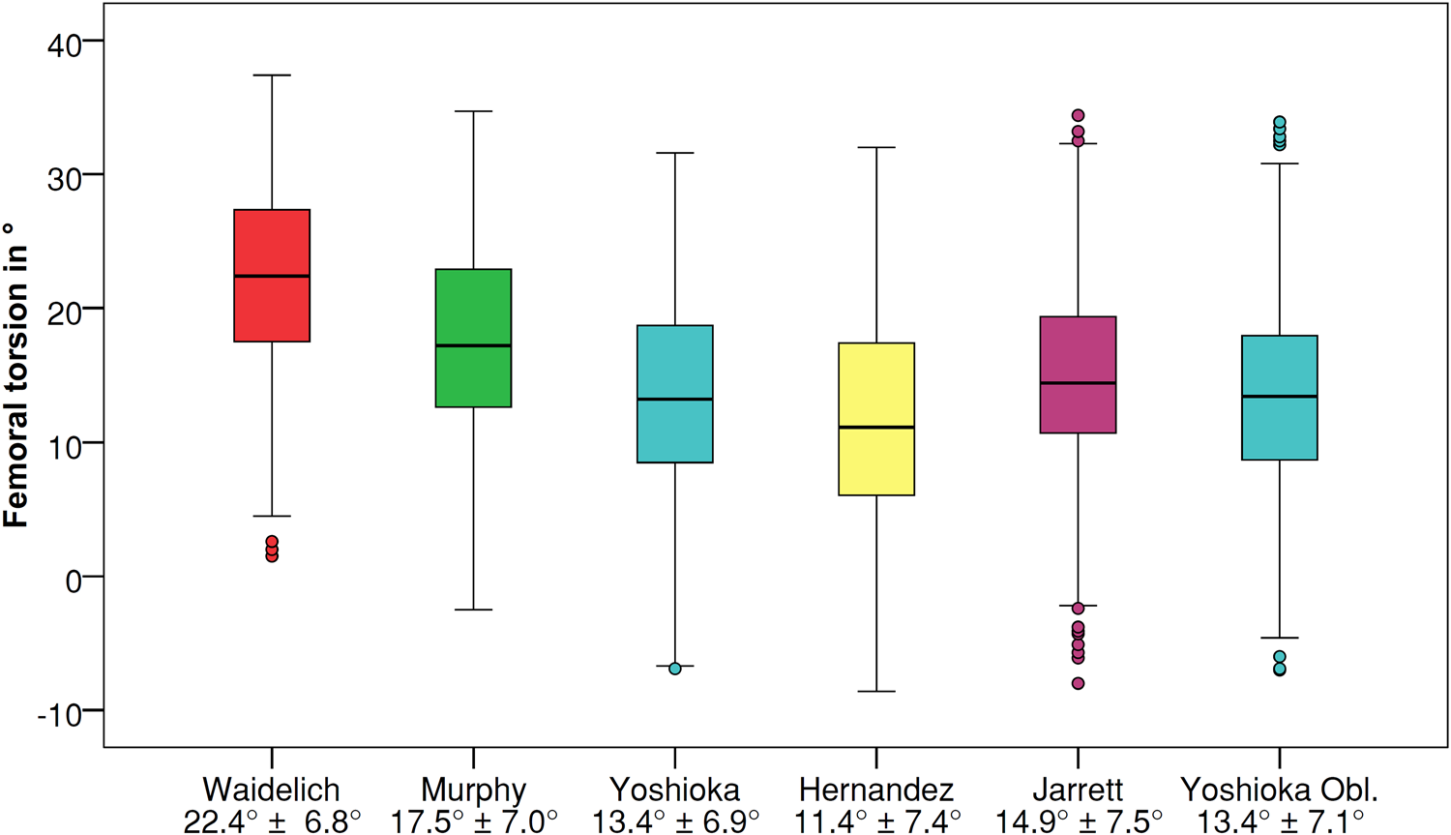
Femoral torsion in degrees measured with different techniques (*x* axis, measurement technique with mean and standard deviation; *y* axis, femoral torsion in degrees (positive values = antetorsion, negative values = retrotorsion; Obl. = Oblique)

Significant differences were observed between pairwise comparisons of the techniques in the values measured for femoral torsion (*P* < 0.001), with the exception of Yoshioka’s technique on transverse and oblique slices (*P* = 1.000). The greatest difference (11°) in the mean value for femoral torsion was found between the Waidelich and Hernandez techniques. These two techniques showed a maximum difference of up to 16° in single femora. All the techniques showed similar standard deviations of approximately 7° (Fig. 3).

### Intraobserver and interobserver agreement

Data for intraobserver and interobserver agreement are summarized in Tables 1 and 2. The techniques all showed good intraobserver and interobserver agreement on the Fleiss et al. score [10]. The mean intraobserver and interobserver differences were small (0.8°–2.9°). Hernandez’s technique showed the largest absolute range for intraobserver and interobserver agreement (11.4° and 13.6°, respectively). The distal axis at the posterior condyles showed good scores, with an ICC of 0.99, a mean intraobserver and interobserver agreement of less than 1°, and maximum variance of 2.6° and 3.6°, respectively (Tables 1, 2).

**Table 1:** Intraobserver agreement

Measurement technique	ICC	Mean	Range
Waidelich	0.88–0.98	0.8°–2.9°	0.0°–8.9°
Murphy	0.95–0.98	1.2°–1.7°	0.0°–6.5°
Yoshioka	0.94–0.97	1.3°–2.0°	0.0°–6.6°
Hernandez	0.94–0.98	1.2°–2.0°	0.0°–11.4°
Jarrett	0.94–0.98	1.3°–2.2°	0.0°–5.5°
Oblique Yoshioka	0.94–0.99	0.9°–1.9°	0.0°–8.1°
Distal angle	0.99	0.5°–0.6°	0.0°–2.6°

*ICC* intraclass coefficient

**Table 2:** Interobserver agreement

Measurement technique	ICC	Mean	Range
Waidelich	0.91–0.92	2.2°–2.6°	0.0°–9.2°
Murphy	0.93	2.1°	0.0°–9.4°
Yoshioka	0.92–0.95	1.7°–2.2°	0.0°–10.4°
Hernandez	0.94–0.96	1.6°–1.9°	0.0°–13.6°
Jarrett	0.92–0.94	2.0°–2.5°	0.0°–8.9°
Oblique Yoshioka	0.96	1.5°–1.6°	0.0°–8.7°
Distal angle	0.99	0.7–0.8°	0.0°–3.6°

*ICC* intraclass coefficient

## Discussion

The most important finding of the present study was that the values measured for femoral torsion showed significant differences (*P* < 0.001) among the measurement techniques using CT scans. Only Yoshioka’s technique on transverse and oblique slices showed comparable values. The maximum differences were observed between the Waidelich and Hernandez techniques, with a maximum difference of up to 16° of femoral torsion for single femora.

The mean values observed in the present study were comparable with the standard values published in the literature for the Waidelich technique (mean 20.4°–24.1°) [21, 23, 24], Yoshioka technique (13.1°) [25], Hernandez technique (12.4°) [15], and oblique slices (15.7°–16.7°) [3, 20, 23]. Thus, it appears to be the case that differences in the values measured for femoral torsion depend more on the measurement technique used than on the specific patient group.

Measuring femoral torsion is important when assessing risk factors for recurrent patellar instability, since increased internal femoral torsion is regarded as a factor that facilitates patellar dislocation [2, 6, 7, 12, 19, 22]. Absolute values for femoral torsion exceeding 15°–25° of internal torsion have been described as representing an indication for derotational osteotomy in patients with recurrent patellar instability [1, 2, 12]. However, these threshold values may lie within the standard range or may even represent external femoral torsion, depending on the measurement technique used (Fig. 1). Absolute threshold values for femoral torsion establishing an indication for derotational osteotomy might be pathological if the Hernandez technique is used, but might also be physiological if the Waidelich technique is used.

Fixing a femoral fracture especially by methods of closed reduction might result in excessive internal or external maltorsion. An increased internal maltorsion seems to be clinically more disabling because of an in-toeing gait than an increased external maltorsion. Measurement of femoral torsion in such cases is, therefore, of major importance to assess the degree of maltorsion. The absolute value of femoral torsion needs to be reflected in regard to the correct measurement technique with its own norm values to plan the correct degree of surgical derotation and not ending up in another malttorsion.

The findings of the present study show that there is a need to use standard values dependent on the measurement technique for femoral torsion. Values for femoral torsion, therefore, have to be interpreted cautiously in relation to the reported threshold values for derotation, as the measurement technique always needs to be taken into account. Radiologic reporting should always include the technique which was used for measurement.

When femoral torsion is being assessed, it is crucial to use a technique that can be repeated with a low level of intraobserver and interobserver agreement. Femoral torsion is measured as the angle between an axis in the proximal and distal parts of the femur. The present study and the literature reports show a low level of intraobserver and interobserver agreement, with a maximum of 3.6° and a mean of 1° for the construction of the distal axis as a tangent to the posterior condyles [16, 18]. It, therefore, appears that the main reason for intraobserver and interobserver differences in measuring femoral torsion is the way in which the proximal axis is constructed. The results of the present study indicate good reproducibility for all of the techniques, with a low mean intraobserver and interobserver agreement of approximately 2°. Despite a high ICC, the technique described by Hernandez showed the greatest maximum intraobserver and interobserver differences (11.4° and 13.6°). Similarly high values have been reported for this technique in the literature [16, 18]. Lower values were noted with the other techniques, a finding that is in agreement with the reported results with the Waidelich [13, 23, 24], Murphy [18], and Jarrett techniques [13] for intraobserver and interobserver agreement. The reason for the higher values with Hernandez’s technique might be that in some cases, the femoral head and neck cannot be visualized adequately on a single slice—especially in the cases in which there is a large femoral neck–shaft angle in the frontal plane (coxa valga) [24]—so that the slice choice for measurement may vary. Superimposed images thus appear to be better for measurement and can be recommended to determine the femoral neck axis.

One limitation of this study is that postmortem femora from elderly patients were used and the sample size was small, so that the study may not provide an adequate basis for deducing standard values for the measurement techniques. In addition, the soft-tissue mass of the thigh and a physiological position on the CT bench were not simulated.

Trauma surgeons as well as radiologists at our institution used the technique described by Waidelich for the measurement of femoral torsion already prior to this study, while the measurement technique was a topic of frequent discussion. With the present study, the currently used method was confirmed as the standard technique in our clinic, because the intra- and interobserver agreement is high and mean values are reported in the literature.

In the conclusion, this study shows that surgeons need to be aware that threshold values for establishing an indication for derotational osteotomy and standard values for femoral torsion always need to be interpreted relative to the measurement technique used, since a pathological value measured with one technique may be physiological using the standard values from another one. With regard to intraobserver and interobserver agreement, techniques that use superimposed images or an oblique image appear to be preferable for measuring femoral torsion. Our institution uses the technique described by Waidelich because of its high intra- and interobserver agreement and the availability of norm values in the literature.
